# Adaptation of perennial flowering phenology across the European range of *Arabis alpina*

**DOI:** 10.1098/rspb.2023.1401

**Published:** 2023-11-22

**Authors:** Jörg Wunder, Andrea Fulgione, Per Toräng, Stefan Wötzel, Michel Herzog, José Ramón Obeso, Yiannis Kourmpetis, Roeland van Ham, Thomas Odong, Marco Bink, Ulla Kemi, Jon Ågren, George Coupland

**Affiliations:** ^1^ Department of Developmental Biology, Max Planck Institute for Plant Breeding Research, Carl-von-Linné-Weg 10, 50829 Cologne, Germany; ^2^ Department of Ecology and Genetics, Evolutionary Biology Centre, Uppsala University, 752 36 Uppsala, Sweden; ^3^ Laboratoire d’Écologie Alpine, LECA, Université Grenoble Alpes, 38000 Grenoble, France; ^4^ Research Unit of Biodiversity (UO-CSIC-PA), Universidad de Oviedo, Campus de Mieres, 33600 Mieres, Spain; ^5^ Biometris, Wageningen University and Research Centre, 6700 AC Wageningen, The Netherlands; ^6^ Laboratory of Bioinformatics, Wageningen University, 6708 PB Wageningen, The Netherlands; ^7^ KeyGene, 6708 PW Wageningen, The Netherlands

**Keywords:** flowering phenology, vernalization, flowering time, life-history, adaptation, drought

## Abstract

Flowering phenology is important in the adaptation of many plants to their local environment, but its adaptive value has not been extensively studied in herbaceous perennials. We used *Arabis alpina* as a model system to determine the importance of flowering phenology to fitness of a herbaceous perennial with a wide geographical range. Individual plants representative of local genetic diversity (accessions) were collected across Europe, including in Spain, the Alps and Scandinavia. The flowering behaviour of these accessions was documented in controlled conditions, in common-garden experiments at native sites and *in situ* in natural populations. Accessions from the Alps and Scandinavia varied in whether they required exposure to cold (vernalization) to induce flowering, and in the timing and duration of flowering. By contrast, all Spanish accessions obligately required vernalization and had a short duration of flowering. Using experimental gardens at native sites, we show that an obligate requirement for vernalization increases survival in Spain. Based on our analyses of genetic diversity and flowering behaviour across Europe, we propose that in the model herbaceous perennial *A. alpina*, an obligate requirement for vernalization, which is correlated with short duration of flowering, is favoured by selection in Spain where the plants experience a long growing season.

## 1. Background

Adaptation of plants to their local environment involves selection on many phenotypic traits, among which flowering phenology can be crucial for plant fitness [1,2]. Life-history theory predicts that reproductive success depends on a trade-off between the age of the plant at flowering (flowering time) and its size [3,4]. Plants that flower soon after germination have low biomass and seed yield, but are more likely to form mature seeds in environments with short growing seasons. By contrast, later flowering plants are larger and have the potential to produce more progeny, but require longer growing seasons to reach maturity. Therefore, the length of the growing season and the time of flowering within the growing season (flowering phenology) can determine reproductive success. Moreover, flowering phenology is responsive to environmental signals such as the seasonal cues of extended cold (vernalization) or day length [2,5]. Appropriate timing of flowering induced by environmental cues can reduce the effect of unfavourable conditions such as frost and drought on seed yield [4]. Therefore, flowering responses to environmental cues can contribute to adaptation to latitude [6,7] and altitude [8,9].

Perennials differ from annuals in the relationship between flowering, development and overall fitness [10,11]. Annual plants more often colonize disturbed environments, flower rapidly to ensure seed production and are short lived [11,12]. By contrast, perennials have a longer lifespan and are typically iteroparous, reproducing multiple times in their lifetime, and during each growing season tend to invest proportionally more in vegetative growth and less in reproductive structures compared with annuals [13]. In contrast to annuals, which senesce and die after flowering, perennials present a more complex flowering behaviour, including the regulation of flowering duration and balancing the allocation of resources between reproduction and growth during and after flowering [14,15]. Also, perennials typically exhibit a longer period of vegetative development prior to flowering, during which they establish greater biomass as well as more axillary meristems. These meristems can acquire different fates, vegetative or reproductive, and they typically sustain multiple reproductive episodes [16,17]. In common with annuals, flowering phenology of perennials is regulated by environmental cues such as vernalization and day length [10,11], and contributes to overall fitness along with other traits such as survival and fecundity [17].

Genetic variation for flowering time is implicated in the adaptation of herbaceous perennials to their local environment. In *Lythrum salicaria* and *Helianthus maximiliani*, northern genotypes flower earlier than southern genotypes in the native range [7,18], and early flowering of *L. salicaria* evolved at higher latitudes during range expansion when introduced into North America [19]. Genetic variation for a floral repressor that confers seasonal flowering patterns exists in the perennial species *Fragaria vesca, Rosa* ssp. [20,21] and a large inversion is associated with perenniality in *Mimulus guttatus* [22]. In perennial Brassicaceae species, orthologues of the *Arabidopsis thaliana FLOWERING LOCUS C* (*FLC*) gene confer a flowering response to vernalization, and influence flowering phenology [14,23,27]. For instance, in *Arabis alpina*, mutants and natural accessions carrying recessive alleles of the *FLC* orthologue *PERPETUAL FLOWERING 1* (*PEP1*) flower without vernalization. In addition, *PEP1* influences flowering duration, so that the mutants flower more prolifically and for a longer duration (perpetually) under controlled conditions compared with plants carrying an active gene [14,23,28]. Geographical variation in flowering time in herbaceous perennials has been described in previous studies [2,7,18,19], but the significance of the duration of flowering to survival in natural populations and how this is related to environmental cues has not been studied. Particularly, the importance of these components of flowering phenology to fitness in natural populations of perennial plants growing in different environments has not been determined.

Here, we test the significance of flowering phenology to adaptation of perennial plants to diverse European environments. Specifically, we test the hypothesis that variation in vernalization response and/or flowering duration contribute to adaptation of European populations of *A. alpina*. First, we use genomic data to study population structure and genetic diversity among the populations, so that these can be compared to the pattern of phenotypic variation. We then examine the flowering phenology of accessions collected across the European range by measuring three flowering traits that capture the flowering phenology of *A. alpina*: the time of flowering onset without vernalization, the time of flowering onset after vernalization and the duration of flowering after vernalization. By comparing the flowering behaviour of different genotypes with their fitness in common gardens, our data indicate that an obligate requirement for vernalization and/or short flowering duration are favoured by selection in southern environments, and we propose that in the perennial life cycle these traits contribute to adaptation to environments exhibiting warmer and longer growing seasons.

## 2. Methods

### (a) Study species

*Arabis alpina* L. (Brassicaceae) shows an Arctic–alpine disjunct distribution and occurs in most European mountain ranges. Seedlings germinate and establish throughout the growing season, but particularly in moist conditions in early summer. The duration from the first open flower to the release of the first mature seeds depends on microclimatic conditions, it may be as fast as six weeks in Spain but in particularly cold microhabitats it may take the entire growing season. Inflorescences are produced from rosettes formed the previous year and continue flowering for several weeks. *Arabis alpina* is a perennial species, is iteroparous and has a mixed mating system. In the present study populations, selfing rates are relatively high [29,30], but self-incompatible populations occur in southern and southeastern Europe [30–32]. Further details are provided in the electronic supplementary material.

### (b) Plant material

Seeds of the 1035 individual plants (accessions) used in this study were either collected from mature open pollinated plants in their natural habitat (*in situ*), defined here as maternal families, or obtained from Genebank Osnabrück (www.genbank-wel.uni-osnabrueck.de) and Botanical Garden Reykjavík (www.grasagardur.is) (electronic supplementary material, table S1). Plants were collected in 44 natural populations across Europe, including three core areas situated in Spain (Cantabrian Mountains; 13 populations), in France (French Alps; 9 populations) and in Norway and Sweden (Scandinavia, 6 populations), and also including widely distributed locations through the eastern Alps, Pyrenees, Canary Islands, Madeira and Germany (electronic supplementary material, table S1). The numbers of accessions per population are listed in electronic supplementary material, table S1. Individual accessions were collected a minimum distance of 1 m from each other to minimize the possibility of sampling genetically identical individuals. Part of this seed collection was previously used to study local adaptation and plant mating system variation [29,30,33,34]. Plants grown from the collected seeds were used for phenotyping and for whole-genome sequencing, while leaves collected *in situ* from a subset of the same plants were genotyped. Genomic analyses by whole-genome sequencing and genotyping are described below.

We used reference accessions with known flowering phenotypes as controls [14,23,28]. The reference accession *Pajares* (originated 30 km from Angliru in population E2) shows the obligate vernalization requirement and short duration of flowering after vernalization that is prevalent in Spanish accessions. The *pep1*-*1* mutant (generated from the accession *Pajares*) carries a mutation in the *A. alpina* orthologue of *FLC*, and is therefore impaired in the vernalization pathway and flowers early and perpetually [14]. The mutant seeds were generated by backcrossing the original mutant [14] twice to *Pajares* wild-type, then self-fertilizing the back-cross-2 heterozygote plants and selecting a homozygous mutant in the progeny. Progeny produced by self-fertilization of this single homozygous mutant were used for the experiments presented.

### (c) Genotyping and analysis of population structure and genetic diversity

Genetic variation and differentiation of *A. alpina* populations were analysed with whole-genome sequence and genotype data. The genome sequences of 27 accessions were obtained and single nucleotide polymorphisms (SNPs) were called as described in the electronic supplementary material. A subset of 253 widely spread, intergenic SNPs identified in the whole-genome sequences was used for genotyping on a Custom GoldenGate SNP-panel. The SNP set was chosen to be informative across the sampled geographical range and/or within regions and populations. Then, DNA extracted from leaves collected *in situ* from a subset of 892 of the same plants used for seed collection was genotyped. More details are provided in the electronic supplementary material.

The SNPs and genome data were used to analyse population structure and genetic diversity among populations to determine whether they followed a pattern similar to or different from phenotypic variation. For analysis of population genetic structure, ‘STRUCTURE’, V2.3.4 [35,36] was used and the Δ*K* method [37] was employed to infer the best number of clusters (*K*). The following data were analysed separately: (i) the complete set of 892 range-wide accessions; (ii) a subsampled set of 450 accessions (50 from each of three populations from each of the three main regions, Spain, the French Alps and Scandinavia) to avoid biases due to sample size differences; (iii) the three main regions independently, to characterize structure within regions. For a model-free visualization of the genetic groups, principal coordinate analysis (PCoA) was also performed [38].

Genetic diversity estimations from genotype data were calculated using Arlequin V3.5.1.2 [39], considering only markers with less than 5% missing data. Nucleotide diversity (average pairwise differences, *π*) was also analysed genome-wide using custom scripts deposited in the Dryad repository at https://doi.org/10.5061/dryad.7wm37pvvm [40].

### (d) Phenotyping experiments

To examine the geographic distribution of flowering traits, we combined phenotyping experiments in greenhouse conditions and at common gardens, and we scored traits *in situ* (described below). The plant material used in each experiment is described in electronic supplementary material, tables S1 and S2. We scored four phenotypes related to flowering behaviour: (i) the onset of flowering without vernalization; (ii) the onset of flowering after vernalization, which was the time from the end of vernalization to the appearance of the first open flower; (iii) the duration of flowering after vernalization, which was the time between appearance of the first and the last open flower; (iv) the proportion of plants flowering per family and per population. In all greenhouse and common-garden experiments, seeds were stratified in cold, moist and dark conditions for 3 days prior to sowing. Plants were organized in a randomized block design, and the reference accession *Pajares* and the *pep1-1* mutant were used as controls because their contrasting flowering behaviours are well defined, as described above in §2b 'Plant material'.

### (e) Greenhouse experiments

To test for differences in flowering behaviour of plants from different geographical regions, flowering traits were scored under controlled conditions in the greenhouse. First, plants across the whole European range were scored for flowering time without vernalization, and then in a second experiment plants from the three main regions were scored in more detail for flowering with and without vernalization treatment (prolonged exposure to cold temperatures).

To assess variation in flowering time across the European range of the species, plants were grown in three replicates of a controlled greenhouse experiment (experiment ID 1, 3, 5; electronic supplementary material, table S2) for at least 26 weeks (26–50 weeks) after germination under long-day conditions (LD: 16 h/8 h day/night cycle, 22°/16°C). Plants represented a range-wide collection of 44 natural populations (electronic supplementary material, table S1), 529 maternal families (2–10 families per population) and 6–8 siblings per family. Plants were scored twice a week for the onset of flowering without vernalization, which is the time between germination and appearance of the first open flower. Because germination was very uniform after seed stratification in cold and moist conditions, we used sowing date as germination date. We assigned plants to three categories: ‘Early-onset’ plants flowered within four months of germination (reflecting the average snow-free period in the Scandinavian and French Alpine habitats). ‘Late-onset’ plants required more than four months from germination to flowering. ‘Not flowering’ plants produced no open flowers during the experiment.

In the second set of greenhouse experiments (experiment ID 2, 4; electronic supplementary material, table S2), we expanded our experimental design by including vernalization treatment, more replicates and more phenotypes, and we focused on the three main regions of the species range. Plants represented the collections in Spain (two populations plus the reference accessions Paj and *pep1-1*), in the French Alps (three populations) and in Scandinavia (five populations), for a total of 10 populations, 187 families and 6–8 siblings per family in the experiment without vernalization, and smaller family sizes in the experiment with vernalization owing to limited space available (experiments 2 and 4 in electronic supplementary material, tables S1 and S2). Plants were grown under two different treatments: (i) experiment without vernalization: plants were grown under long days (LD: 16 h/8 h day/night cycle, 22°/16°C) for 44 weeks without vernalization; (ii) experiment with vernalization: plants were grown in long days for six weeks, then vernalized for about 14 weeks (short days, SD: 8 h/16 h day/night cycle, 4°C), and then transferred back into LD for 19 weeks. The vernalization treatment is designed to roughly follow the changes in photoperiod through the year, with long days in the warm season when seeds germinate, short days in the autumn and winter (during vernalization), and again long days in the warm season the following year.

### (f) Common-garden experiments in the field

To assess the flowering behaviour and performance of plants from each of the main regions under natural conditions, we performed reciprocal transplant experiments at sites in Spain (Angliru, N43.22969°, W005.93873°, 1525 m), the French Alps (Lautaret, N45.03646°, E06.3998°, 2100 m), and Scandinavia (Geargevággi N68.4100°, E18.3195°, 950 m), close to or within native populations (experiment ID 7–10; electronic supplementary material, table S2). In addition, we grew plants in a common garden in Germany (MPIPZ, N50.9563°, E06.8611°, 55 m) outside the natural range (experiment ID 11; electronic supplementary material, table S2). Plants were pre-grown in small plugs for about 4 weeks in a greenhouse to ensure high establishment success. The seedlings were then transplanted into natural soil at the experimental sites in spring. Apart from watering on the first few days after planting, the plants grew under natural environmental conditions and in native soil. The three main regions of origin were represented at each site, with two populations from Spain plus the reference accessions Paj and *pep1-1*, three populations from the Alps and five from Scandinavia (10 populations in total), about 15 families per population and 10 siblings per family (electronic supplementary material, tables S1 and S2). We scored individual plants near the end of the growing season in the year of planting and the following year, for survival and whether they had flowered (inferred by whether plants bore fruits). We defined the growing season as the number of days between the mid-points of the first and last snow-free, 5-day period with mean temperature greater than 5°C.

At the Spanish site, we performed an additional experiment (experiment ID 6; electronic supplementary material, table S2) focused on studying the relationship between flowering phenology and fitness in more detail in this environment. We used the reference accessions Paj and *pep1-1* as the main contrast because they differ in flowering behaviour but share the same genomic background. We also used accessions from two Spanish populations with 42 families and around 14 siblings per family (electronic supplementary material, tables S1 and S2) as representatives of typical Spanish populations. We counted the number of rosettes on each plant as a measure of vegetative performance, and the number of fruits (siliques) as a measure of fecundity (electronic supplementary material).

### (g) Observations *in situ*

Plants in Spain and the Alps were followed *in situ* to determine whether the flowering phenology, particularly perpetual flowering, predicted from the greenhouse experiments occurred at the natural sites. In seven natural populations, three in Spain and four in the Alps, individual plants were labelled, visited two to five times per year over several years, photographed and scored for whether or not they were flowering. Each year one visit was included close to the end of the growing season between 15 September and 8 October, to estimate the proportion of plants with open flowers until the end of the growing season, or perpetually flowering (electronic supplementary material).

### (h) Weather data

We measured *in situ* air temperature and we obtained precipitation data from nearby public weather stations (more detail in electronic supplementary material).

### (i) Statistical analyses

Statistical analyses were conducted in R, version 3.6.2 [41]. To model whether plants flowered or not in greenhouse conditions without vernalization, and to test for the effect of vernalization on flowering in greenhouse conditions and in common-garden experiments in the field, we used a generalized linear mixed-effect model (glmm) with a binomial family and logit link function, using the R package ‘lme4′, function *glmer* (general formula: Binomial_outcome∼region + (1|population:family); with or without the addition of fixed factors *vernalization* and *experimental site*). However, for flowering without vernalization, because of *quasi-complete separation* of the outcome by a predictor variable, the maximum-likelihood estimates of parameters do not exist (more detail in electronic supplementary material) and this method could not be used [42]. Therefore, we adopted a different approach and used the asymptotic two-sample Fisher–Pitman permutation test implemented in the R package ‘coin’, function *oneway_test*.

To model the time of flowering onset without vernalization, and the duration of flowering after vernalization we fitted a Cox proportional-hazards model implemented in the R package ‘survival’, function *coxph* (formula: Surv(Flowering_onset or duration, Flowering_binomial)∼region).

To test for differences in survival in common-garden experiments, we used Fisher's exact test implemented in the R function *fisher.test*. To test for correlations between traits, we used Spearman's rank correlation when relationships were nonlinear and the assumption of normality was not appropriate for residuals, and in other cases the Pearson's product–moment correlation. Plants that did not flower were excluded from tests involving flowering time. The tests for correlations were implemented in the R package ‘Hmisc’, function *cor.test*. To test for homogeneity of group variances, we applied the Brown–Forsythe test implemented in the R package ‘onewaytests’, function *bf.test*. To test for differences in flowering duration between accessions that never flowered without vernalization and all other accessions, in flowering time between perpetually flowering plants and all other plants, and to test for differences between *pep1-1* and *Pajares* in survival, vegetative and reproductive performance, we conservatively used the nonparametric Wilcoxon rank-sum test with continuity correction implemented in the R function *wilcox.test*, to limit assumptions about the distribution of residuals.

## 3. Results

### (a) Population genetic structure of European *Arabis alpina*

**Figure 1.**
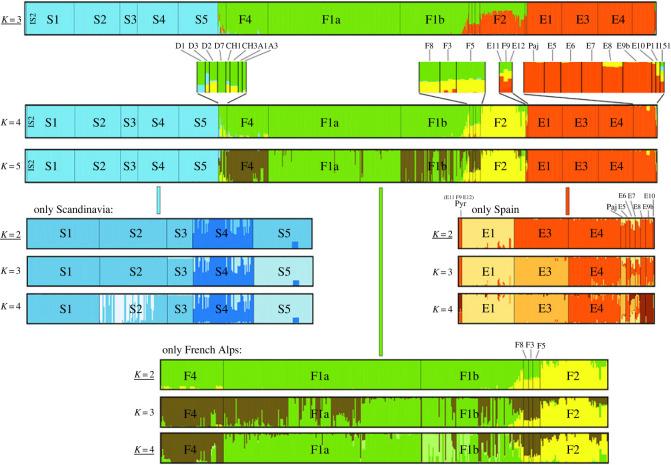
Population structure of European *Arabis alpina*. Inferred population structure based on 253 single nucleotide polymorphisms (SNPs) and 892 individuals from across Europe (top), and independently for each of the three major regions, Scandinavia, French Alps and Spain (below). Best *K* according to the Δ*K* method is underlined.

A broad representation of population structure across the European range was obtained by assessing genetic variation and differentiation at 253 SNPs in 892 accessions collected across 36 populations. These accessions represent the major areas of the European range: northern Spain, the Alps, Scandinavia, the Pyrenees, Canary Islands, Madeira and several German populations (electronic supplementary material, table S1). Bayesian modelling using STRUCTURE [35,36] identified three major regions, Spain, the Alps (including the German relict populations) and Scandinavia (figure 1). Increasing the number of clusters and treating regions separately first differentiated the regions then populations within regions, revealing hierarchical population structure. The separation across regions is as clear in a subset of the samples designed to avoid biases due to sample size differences (50 individuals from each of three populations in each region for a total of 450 individuals) as in the entire dataset (electronic supplementary material, figure S1), and it reveals high differentiation among populations (average *F*_st_ across populations: 0.38 in Spain, 0.46 in the Alps and 0.62 in Scandinavia, table 1*a*).

**Table 1.** Genetic diversity and population structure of European *Arabis alpina*. SNPs, single nucleotide polymorphisms.

population …	Spain				France				Scandinavia				all
	E1	E3	E4	all E	F1a	F2	F4	all F	S1	S4	S5	all S	total
(*a*) based on 450 individuals and 253 SNPs; calculations were performed with Arlequin V3.5.1.2 [49]													
no. individuals	50	50	50	150	50	50	50	150	50	50	50	150	450
no. loci	250	245	243	244	241	244	236	240	244	244	241	243	242
no. SNPs	44	83	100	113	62	79	68	113	9	23	7	39	226
nucleotide diversity, *π* (%)	3.3	10.4	13.7	13.0	7.8	8.2	8.3	13.0	1.2	3.9	0.8	4.0	67
var. between pop., *F*_st_^a^	—	—	—	0.38	—	—	—	0.46	—	—	—	0.62	0.42
var. within pop.^a^	—	—	—	0.4	—	—	—	0.4	—	—	—	0.35	—
var. within individuals^a^	—	—	—	0.22	—	—	—	0.14	—	—	—	0.03	—
observed heterozygosity^a^	0.03	0.1	0.14	0.07	0.13	0.04	0.03	0.04	0.02	0.03	0.03	0.01	—
expected heterozygosity^a^	0.19	0.31	0.33	0.28	0.31	0.26	0.29	0.28	0.33	0.41	0.28	0.26	—
inbreeding coefficient, *F*_IS_^a^	0.82	0.67	0.59	0.65	0.56	0.8	0.88	0.75	0.93	0.93	0.91	0.93	—
(*b*) based on whole-genome data from 27 individuals; nucleotide diversity calculated with custom scripts (see Methods).													

	**Spain**			**France**			**Scandinavia**					**all**	
**population …**	**E3**	**E4**	**all E**	**F1a**	**F2**	**all F**	**S1**	**S2**	**S4**	**S5**	**all S**	**total**	
no. individuals	4	4	8	4	4	8	3	3	3	2	11	27	
nucleotide diversity, *π*	1.40 × 10^−3^	2.23 × 10^−3^	2.09 × 10^−3^	1.62 × 10^−3^	1.22 × 10^−3^	3.09 × 10^−3^	1.14 × 10^−4^	5.44 × 10^−5^	9.01 × 10^−5^	9.01 × 10^−5^	1.48 × 10^−4^	4.74 × 10^−3^	

^a^Calculations performed with 10 000 permutations; all results significant with *p* < 0.00001.

### (b) Flowering time without vernalization is geographically structured among European *Arabis alpina* accessions

**Figure 2.**
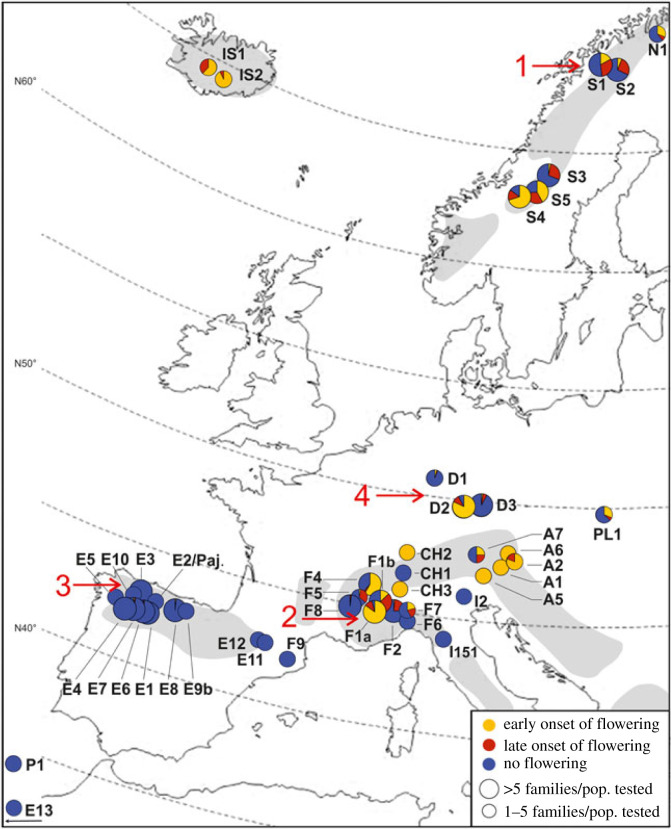
Geographical distribution of flowering time of European *Arabis alpina* when grown without a vernalization treatment. Each circle represents a population that was tested for the time of flowering onset under controlled greenhouse conditions without vernalization (4002 tested individuals). Early flowering is defined as flowering less than four months after germination; late flowering, as flowering in more than four months; not flowering was scored until the end of the experiments. Red numbers represent the experimental sites (1, Geargevággi; 2, Lautaret; 3, Angliru; 4, MPIPZ). The grey areas indicate the natural distribution range according to http://linnaeus.nrm.se/flora/.

We assessed variation in the time from germination to flowering (flowering time) using accessions collected across the European range of the species in controlled greenhouse conditions without vernalization. The accessions used in each experiment are described in electronic supplementary material, tables S1 and S2. Accessions from the majority of tested regions harboured variation in flowering time, with plants flowering early, late or not at all without vernalization (figure 2 and electronic supplementary material, table S3). Plants that flowered early without vernalization were collected in almost all populations in central and northern Europe (Germany, the French and eastern Alps, Iceland, Scandinavia). Populations in the French Alps and Scandinavia included plants that flowered early, late or not at all, and in several cases all of these flowering phenotypes were found within single populations (e.g.: F1b, S4 and S5). By contrast, all individuals from each of the 10 populations collected in northwestern Spain as well as the three populations collected in the Pyrenees did not flower under these conditions, indicating that this non-flowering phenotypic class predominates in Spain (figure 2 and electronic supplementary material, table S3). There was thus a stark contrast between Spanish populations, which exclusively show a non-flowering phenotype under controlled conditions without vernalization, and populations from other parts of Europe, which harbour considerable variation in flowering time, including extreme early flowering plants.

### (c) Effect of vernalization on flowering behaviour of *Arabis alpina* accessions under controlled greenhouse conditions

**Figure 3.**
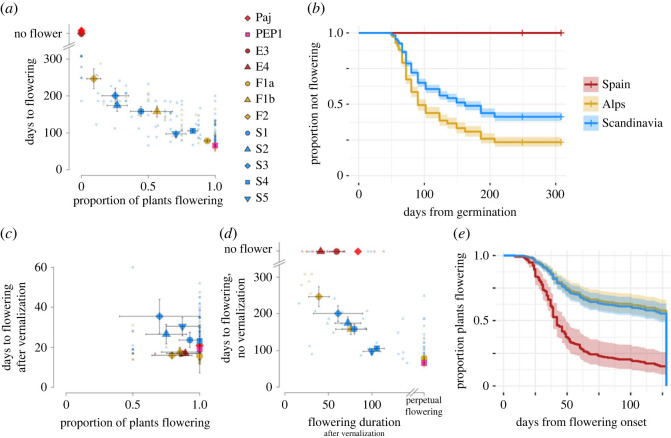
Flowering behaviour of non-vernalized and vernalized *A. alpina* plants in controlled greenhouse conditions. (*a*) Relationship between the proportion of plants flowering (*x*-axis) and flowering time (*y*-axis) without vernalization. No Spanish populations or families flowered without vernalization (jittered on the top left, no flower). (*b*) Proportion of non-flowering plants (growing vegetatively) without vernalization, as a function of time since germination and region of origin (Spain, the Alps and Scandinavia), modelled with a Cox proportional-hazards model. (*c*) Proportion of plants flowering and flowering time in the experiment with vernalization (exposure to 4°C for about 14 weeks in short-day conditions). Note the different scale on the *y*-axis, where zero represents the first day after vernalization (132 days after sowing). (*d*) Relationship between flowering duration after vernalization (*x*-axis) and flowering time without vernalization (*y*-axis). Populations and families that did not flower without vernalization (mainly Spanish) are shown at the top of the panel. Populations and families that did not arrest flowering (perpetually flowering) are shown to the right in the panel. Large symbols represent reference accessions and population averages with 95% CI as error bars. Small symbols represent family averages. (*e*) Proportion of plants that continue flowering as a function of time since flowering onset after vernalization (duration of flowering) and region of origin, modelled with a Cox proportional-hazards model. The legend of (*a*) is in common with (*c*) and (*d*); the legend of (*b*) is in common with (*e*).

The analysis of flowering time described above was extended by testing the effect of vernalization on flowering, and by scoring the duration of flowering after vernalization, with a focus on the three main regions of the species range (Spain, the Alps and Scandinavia). First, the onset of flowering under controlled greenhouse conditions without vernalization was tested as a negative control for the vernalization treatment. This experiment was similar to the one described above (§3b) but was performed with a larger set of families per population (figure 3*a* and electronic supplementary material, table S4). First, we analysed the proportion of plants flowering across families and regions of origin. Consistent with the findings reported above, neither the reference accession *Pajares* from Spain nor any other plant from Spanish populations (305 individuals from 40 families from sites E3 and E4) flowered under these conditions. The *pep1-1* mutants, on the other hand, all flowered, consistent with its loss of a requirement for vernalization [14]. Based on permutations of the region of origin, the uniform behaviour of Spanish families differed strongly from families collected in the French Alps (Fisher–Pitman permutation test: *Z* = −7.5341, *p* = 4.918 × 10^−14^) and Scandinavia (*Z* = 7.2113, *p* = 5.542 × 10^−13^). French families differed only marginally from Scandinavian families, and both varied markedly in the proportion of flowering plants (between 9 and 95% of plants from the French Alps flowered across populations, and between 17 and 82% of plants from Scandinavia; figure 3*a* and electronic supplementary material, table S4). Because of *quasi-complete separation* of the outcome (flowering traits) by a predictor variable (factor *region*, level *Spain*), a glmm approach failed to converge in this case (more information in electronic supplementary material).

In terms of the time of flowering onset, as expected, the *pep1-1* mutants all flowered early in the experiment (mean flowering time ±95% CI: 66 ± 3 days after germination). Families from Spain did not flower, and families from the French Alps and Scandinavia varied greatly in the time of flowering onset (French Alps: between 79 ± 4 and 247 ± 27 days from germination across populations; Scandinavia: between 98 ± 8 and 200 ± 21 days from germination; figure 3*a*). Based on fitting a Cox proportional-hazards model to the time of flowering onset, families from Scandinavia had a 39% lower hazard of flowering compared with families from the Alps (*z* = −7.495; *p* = 6.62 × 10^−14^; figure 3*b* and table 2*a*). In spite of the even more extreme flowering phenology of Spanish families, which did not flower, this approach failed to detect a significant difference due to *quasi-complete separation* (more information in electronic supplementary material). If we conservatively assume that all non-flowering plants flowered the day after the experiment ended, we identify a significant difference for Spanish families, which have a 63% lower hazard of flowering compared with families from the Alps (*z* = −13.921; *p* < 2 × 10^−16^; table 2*b*).

**Table 2.** Parameters of Cox proportional-hazards models fitted to the time to flowering onset and to flowering duration.

parameter	coefficient	s.e.	z-value	Pr(>|*z*|)
(*a*) model fitted to the time between germination and flowering onset				
region_ES	−18.87	596.1	−0.032	0.975
region_NO	−0.494	0.066	−7.495	6.62 × 10^−14^
(*b*) model fitted to the time between germination and flowering onset, assuming that non-flowering plants all flowered the day after the experiment ended (308 days)				
region_ES	−0.997	0.072	−13.921	<2 × 10^−16^
region_NO	−0.325	0.054	−6.028	1.66 × 10^−09^
(*c*) model fitted to the time between flowering onset and flowering arrest (flowering duration)				
region_ALP	−1.23	0.184	−6.702	2.06 × 10^—11^
region_NO	−1.17	0.173	−6.731	1.69 × 10^−11^

Interestingly, across all regions, the proportion of plants in a population that flowered without vernalization was negatively correlated to the mean time of flowering onset of the population (Pearson's correlation coefficient among population means: *r*_7_=−0.972, *p* = 1.203 × 10^−5^; among family means: *r*_191_ = −0.859, *p* < 2.2 × 10^−16^; figure 3*a*). Therefore, individuals in populations with a high proportion of flowering plants tended on average to flower earlier than those in populations with a high proportion of plants that never flowered.

Next, we documented flowering behaviour among plants that had been subjected to vernalization. Exposure to 4°C for about 14 weeks under short-day conditions induced flowering in the vast majority of plants (70–100% of plants across populations flowered during the 39 weeks of the experiment; glmm estimated effect = 5.86, s.e. = 0.488, *z* = 12.03, *p* < 2 × 10^−16^; figure 3*c* and electronic supplementary material, table S5). Flowering mostly took place soon after vernalization (populations flowered on average between 15 and 36 days after vernalization), resulting in a 37-fold reduction in the variance in the time to flowering (from $\sigma_{\mathrm{not}\;\mathrm{vernalized}}^{2}=3423.8$ to $\sigma_{\mathrm{vernalized}}^{2}=93.3$ days^2^; Brown–Forsythe test of variance: *F*_1,1157_ = 491.5, *p* = 4.92 × 10^−91^).

Although vernalization strongly reduced variation in the proportion of plants flowering and their time to flowering, variation in the duration of flowering after vernalization was extensive (figure 3*d*). Spanish families flowered for a short time (mean duration of flowering ±95% CI, population E4: 41 ± 4 days, population E3: 60 ± 8 days), while French Alpine and Scandinavian families exhibited both short and long durations of flowering (French between 40 ± 12 and more than 128 days; Scandinavian between 61 ± 28 and 106 ± 9 days), with some plants effectively flowering perpetually until the end of the experiment. Based on fitting a Cox proportional-hazards model to the time between flowering onset and arrest, French and Scandinavian families had a significantly lower hazard (respectively, 71 and 69%) to arrest flowering compared with Spanish families (level France: *z* = −6.702, *p* = 2.06 × 10^−11^; level Scandinavia: *x* = −6.731, *p* = 1.69 × 10^−11^; figure 3*e* and table 2*c*).

Strikingly, the time to flowering without vernalization was negatively correlated to the duration of flowering after vernalization (Spearman's correlation coefficient among population means: *r*_s_ = −0.8501, *p* = 4.608 × 10^−4^; among family means: *r*_s_ = −0.7164, *p* = 2.287 × 10^−16^; figure 3*d*). At the one extreme, families that never flowered without vernalization flowered for a shorter duration after vernalization (*n* = 35; average flowering duration ±95% CI: 53 ± 8 days) compared with all other families (*n* = 61; 104 ± 10 days; Wilcoxon rank-sum test, *W* = 373, *p* = 6.095 × 10^−8^). At the opposite extreme, families that flowered perpetually after vernalization, flowered earlier without vernalization (*n* = 35; average time of flowering onset ±95% CI: 101 ± 14 days) compared with all other families (*n* = 61; 282 ± 26 days; Wilcoxon rank-sum test, *W* = 164, *p* = 1.827 × 10^−12^). Taken together, flowering behaviour under controlled conditions of *A. alpina* accessions collected across the species range showed substantial variation in the time to flowering without vernalization and in the duration of flowering after vernalization.

### (d) Flowering behaviour in common-garden experiments in the field

We assessed flowering behaviour of *A. alpina* populations in reciprocal transplant experiments at natural field sites in Spain, the French Alps and Scandinavia, and in a common-garden experiment in Germany (electronic supplementary material, table S2). At each site, we recorded precipitation, temperature and snow coverage (electronic supplementary material, figures S2, S3 and table S6).

**Figure 4.**
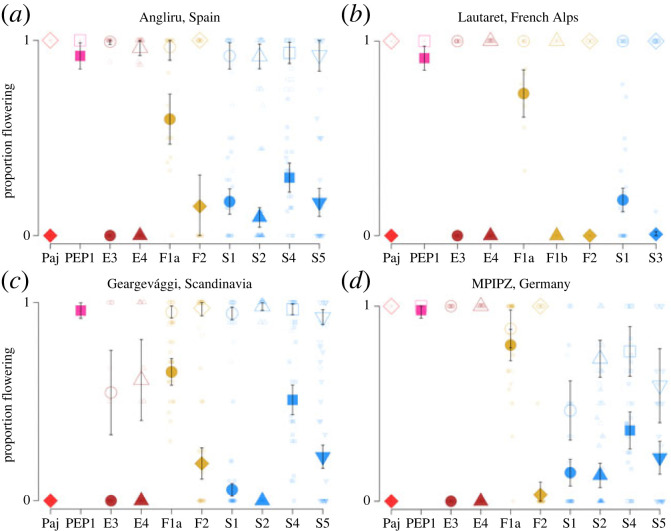
Proportion of flowering plants in common-garden experiments in the field. Proportion of plants flowering before and after the first winter (respectively, full and empty symbols). Results from plantings at (*a*) Angliru, Spain, (*b*) Lautaret, the French Alps, (*c*) Geargevággi, Scandinavia and (*d*) MPIPZ, Germany (outside the natural distribution). Large symbols represent reference accessions and population averages with 95% CI as error bars. Small symbols represent family averages.

Consistent with observations under controlled greenhouse conditions, during the first year of the experiment none of the Spanish individuals flowered at any of the experimental sites, while a significantly greater proportion of French Alpine and Scandinavian plants flowered (e.g. up to 80% of F1a plants, up to 51% of the Scandinavian S4 plants and 98% of the *pep1-1* mutants, figure 4; electronic supplementary material, table S4; glmm estimated effect, level France: 6.54, s.e. = 0.669, *z* = 9.78, *p* < 2 × 10^−16^; level Scandinavia: 4.57, s.e. = 0.622, *z* = 7.36, *p* = 1.91 × 10^−13^; Fisher–Pitman permutation test, range of *p*-values between Spanish and non-Spanish families across sites: 4.71 × 10^−3^–5.37 × 10^−10^). Further, the proportion of plants flowering increased markedly in the second year in all populations (glmm estimated effect = 5.97, s.e. = 0.137, *z* = 44.57, *p* < 2 × 10^−16^; percentile increase in the proportion of plants flowering between 58 and 80% across sites; figure 4 and electronic supplementary material, table S5), again consistent with our observation that vernalization induces flowering in greenhouse conditions.

**Figure 5.**
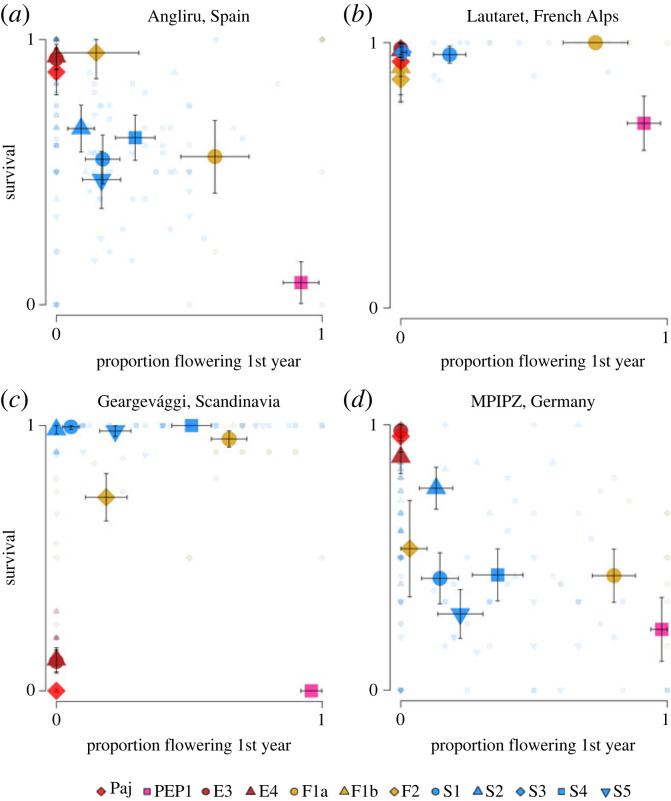
Relationship between the proportion of plants flowering the first year and survival in common-garden experiments in the field. In each panel, the *x*-axis shows the proportion of plants flowering in the year of planting, the *y*-axis shows the proportion of plants surviving into the second year, one year after planting. Results from plantings at (*a*) Angliru, Spain, (*b*) Lautaret, the French Alps, (*c*) Geargevággi, Scandinavia and (*d*) MPIPZ, Germany (outside the natural distribution). Large symbols represent reference accessions and population averages with 95% CI as error bars. Small symbols represent family averages.

Interestingly, the relationship between the proportion of plants flowering in the first year and survival to the next season, a major fitness component, varied across experimental sites. At the high-latitude site in Scandinavia and the high-altitude site in the French Alps, the proportion of plants flowering was not negatively correlated with survival (Pearson's correlation coefficient among population means in Scandinavia: *p* = 0.970; in France: *p* = 0.164; among family means in Scandinavia: *r*_157_ = 0.381, *p* = 7.09 × 10^−7^; in France: *p* = 0.728; figure 5). At the French site, survival was uniformly high, whereas at the Scandinavian site, all accessions except those from Spain had a high survival (figure 5). At the Spanish and German sites, however, the proportion of plants flowering in the first year was negatively correlated with survival to the next year (Pearson's correlation coefficient among population means in Spain: *r*_8_=−0.842, *p* = 2.23 × 10^−3^; in Germany: *r*_8_=−0.696, *p* = 2.55 × 10^−2^; among family means in Spain: *r*_143_=−0.204, *p* = 1.40 × 10^−2^; in Germany: *r*_159_=−0.376, *p* = 8.95 × 10^−7^; figure 5). Notably, in both Spain and Germany, the difference in survival between *Pajares* and *pep1-1* was greater than in France and Scandinavia (Fisher's exact test: in Spain, odds ratio = 0.014, *p* < 2.2 × 10^−16^; in Germany, odds ratio = 0.015, *p* < 5.94 × 10^−14^; in France, odds ratio = 0.178, *p* = 1.88 × 10^−4^; in Scandinavia, odds ratio = 1, *p* = 1). This suggests that in lower latitude and altitude environments, a stronger requirement for vernalization and late flowering is associated with a greater probability to survive to the next year.

### (e) Flowering behaviour *in situ*

In addition to the analyses under controlled conditions and at experimental sites, the duration of flowering was also scored in natural populations, *in situ*, in the French Alps (300 plants) and in Spain (about 150 plants; electronic supplementary material, table S7). While all plants flowered soon after snow melt, none of the Spanish plants bore open flowers late in the growing season, consistent with the short flowering duration following vernalization observed under controlled conditions (figure 3*d*). By contrast, the behaviour of French Alpine plants was more variable, with a fraction of plants still flowering late in the season (between 5 and 40% across populations, averaged across years, electronic supplementary material, table S7). This was particularly prominent in population F1a, with up to 66% of plants still flowering late in the season in 2011, and consistent with the longer duration of flowering of French plants observed in the greenhouse (figure 3*d*). Thus, the flowering behaviour of plants *in situ* in Spain and the French Alps was consistent with their different behaviour in greenhouse conditions and in common-garden experiments in the field.

### (f) Spanish *Arabis alpina* populations show high genetic variation and differentiation

The assessment of flowering behaviour across the European range of *A. alpina* raises the question why populations from the Iberian Peninsula uniformly show an obligate requirement for vernalization and short duration of flowering. Whether this phenotypic homogeneity reflects broad genetic uniformity was assessed by analysing genetic variation and differentiation within and among regions, using both genotype data and whole-genome sequences. First, based on genotype data, the Spanish genomes had as much nucleotide diversity as those from the French Alps, and both regions harboured higher diversity than Scandinavia (*π*_Spain_ = 0.130; *π*_Alps_ = 0.130; *π*_Scandinavia_ = 0.040; table 1*a*). Consistently, Spain and the French Alps harboured similar diversity within populations (range of *π* within populations, respectively: 0.033–0.137 and 0.078–0.083) and within individuals (range of heterozygosity (*H*), respectively: 0.03–0.14 and 0.03–0.13, range of inbreeding coefficient (*F*_IS_: 0.59–0.82 and 0.56–0.88) in contrast to the low diversity within Scandinavian populations (range of *π*: 0.008–0.039) and individuals (range of *H*: 0.02–0.03; range of *F*_IS_: 0.91–0.93, table 1*a*). Analysis of 187 Spanish accessions from 10 populations with STRUCTURE and PCoA identified clear population structure in that region (figure 1; electronic supplementary material, figure S4). Interestingly, the three populations tested from the Pyrenees (F9, E11 and E12; electronic supplementary material, table S1) showed the signature of admixture between Spanish and French Alpine clusters (figure 1), but had the flowering behaviour typical of Spanish populations (figure 2). Finally, genome-wide data on a smaller set of samples supported similar levels of nucleotide diversity in Spain and the French Alps and lower diversity in Scandinavia (*π*_Spain_ = 0.209%; *π*_Alps_ = 0.309%; *π*_Scandinavia_ = 0.015%, table 1*b*). In conclusion, Spanish samples are as genetically diverse as the French Alpine and strikingly more so than the Scandinavian populations. Although genetic variation for ecologically relevant traits is not always related to overall patterns of genomic variation [43,44], these results exclude the possibility that phenotypic homogeneity of Spanish families (figure 3*a,d*) is due to overall low genetic diversity.

### (g) The obligate requirement for vernalization and/or short flowering duration provide a fitness advantage in Spain

**Figure 6.**
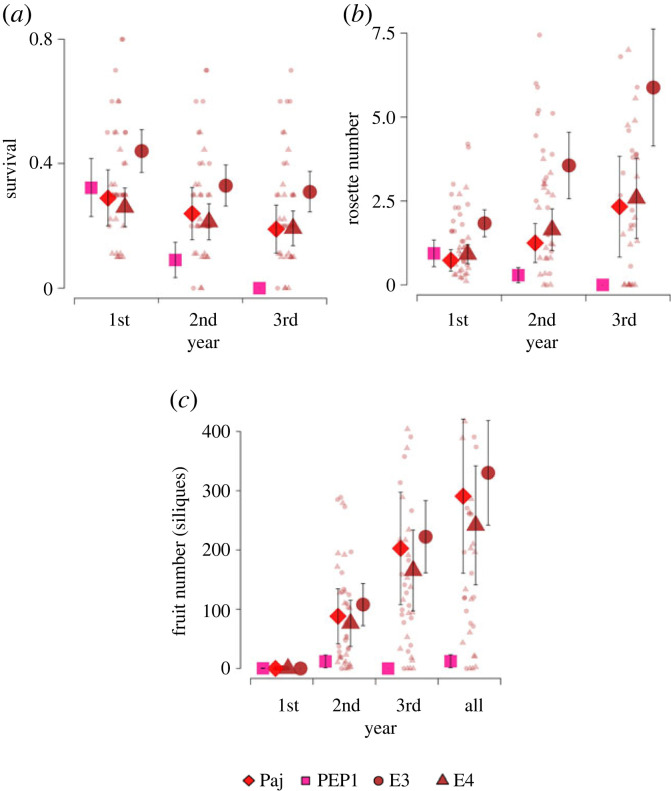
Reproductive success and vegetative performance of the *pep1-1* mutant compared with its wild-type *Pajares* and accessions from Spanish populations in the common-garden experiment at Angliru, Spain. Accessions were planted in May 2010, and scored in September for 3 years. (*a*) Proportion of plants alive. (*b*) Number of vegetative rosettes (greater than 1 cm). (*c*) Number of fruits per plant in individual years and in total. Large symbols represent reference accessions (Paj, *n* = 100; PEP1, *n* = 99) and the averages for 22 families from each of the natural populations E3 and E4 (200 and 192 plants scored; electronic supplementary material, table S8) with 95% CI as error bars. Small symbols represent family averages.

To test the hypothesis that the obligate vernalization requirement and/or shorter duration of flowering prevalent in Spanish populations provide a fitness advantage in the local environment, we compared fitness (survival and fecundity) and vegetative growth (rosette number) of two Spanish genotypes differing in flowering behaviour in a common-garden experiment in Spain (Angliru within population E3; electronic supplementary material, figure S5). We used the Spanish reference accession *Pajares*, and the *pep1*-*1* mutant in *Pajares* background as control genotypes that show contrasting flowering behaviour. For comparison, we also scored 200 and 192 individuals, respectively, from 22 and 20 families from the E3 and E4 Spanish populations (electronic supplementary material, table S8). During the first year, the *pep1-1* mutant performed similarly to the wild-type *Pajares* in terms of survival (Wilcoxon rank-sum test, *W* = 5114.5, *p* = 0.613; figure 6*a*) and rosette number (*W* = 5034.5, *p* = 0.796; figure 6*b*). Unlike *Pajares* and the other natural accessions, the *pep1-1* mutant flowered during the first year, but its fruit production was low, on average 0.313 ± 0.309 fruits per plant (*N* = 32), probably owing to the small size of the plant at this early stage and the onset of winter precluding further fruit production. In the following years, the performance of the *pep1-1* mutants deteriorated compared with *Pajares* and the other natural populations (figure 6; electronic supplementary material, figure S6 and table S8). Significantly lower proportions of the *pep1-1* plants survived during the second and third years of the experiment compared with *Pajares* (2011: *pep1-1* = 9%; *Pajares* = 24%; *W* = 4212, *p* = 4.827 × 10^−3^; 2012: *pep1-1* = 0%; *Pajares* = 19%; *W* = 4009, *p* = 5.459 × 10^−6^), and on average they produced fewer vegetative rosettes (2011: *pep1-1* = 0.3; *Pajares* = 1.2; *W* = 4124.5, *p* = 1.687 × 10^−2^; 2012: *pep1-1* = 0.0; *Pajares* = 2.3; *W* = 3977, *p* = 2.948 × 10^−4^) and fewer fruits (2011: *pep1-1* = 12.0; *Pajares* = 88.1; *W* = 4159, *p* = 2.661 × 10^−3^; 2012: *pep1-1* = 0.0; *Pajares* = 202.6; *W* = 3960, *p* = 3.029 × 10^−6^). Total silique production at the end of the three-year experiment, which is a proxy for overall reproductive success, was 23.7-fold higher for *Pajares* compared with *pep1-1* (*pep1-1* = 12.3 fruits; *Pajares* = 290.8 fruits; *W* = 4238, *p* = 9.060 × 10^−3^) and, respectively, 26.9-fold and 19.7-fold higher for the E3 and E4 natural populations compared with *pep1-1* (figure 6*c*; electronic supplementary material, table S8).

Therefore, in the Spanish environment, the obligate requirement for vernalization, which is correlated with short duration of flowering and prevalent in Spanish accessions, provides a clear fitness advantage over the multiple years of the perennial life cycle.

## 4. Discussion

### (a) Significance of early flowering caused by loss of a requirement for vernalization

In natural environments, all *A. alpina* plants are exposed to vernalization and most start flowering in spring two to three weeks after snow melt. Early flowering without exposure to vernalization would only enhance fitness in the first year after germination, prior to exposure to the first winter. Some French plants and the *pep1-1* mutant grown at the experimental sites in near-natural conditions did indeed flower during the first year prior to winter. However, the plants were planted as established rosettes, which were thus larger and older than plants that established naturally through *in situ* seed germination and seedling growth. Therefore*,* flowering prior to the first exposure to vernalization is likely to be responsible for the formation of few to no seeds in natural environments. Furthermore, flowering in the first year did have a negative effect on survival, particularly at the Spanish and German experimental sites, and this might contribute to the selection for an obligate vernalization requirement in Spain.

Variation in the requirement for vernalization was also correlated with the duration of flowering after vernalization. These two traits are conferred by the same genetic mechanisms [14], and were highly correlated in natural populations. Therefore, their relative significance cannot easily be distinguished. Duration of flowering is likely to have an effect on seed yield, and genetic mechanisms to actively terminate flowering exist in many perennials. In the tree species *Populus deltoides*, the ability to restrict flowering to spring divides the life cycle into periods when flowering is induced and periods of vegetative growth [45]. In herbaceous perennials such as *F. vesca, Rosa* ssp. and *A. alpina*, genetic mechanisms that actively restrict the duration of flowering have been described by identifying mutants that flower continuously [14,20,21,46]. Active termination of flowering might provide several advantages to herbaceous perennials. First, by restricting flowering to spring, plants might avoid allocating resources to flowers with a low probability of successful fruit development because of lower water availability in the summer and reduced time available to form mature fruits before the end of the growing season. Instead, resources can be allocated to functions increasing likelihood of survival. Second, in most herbaceous perennials, the transition of a meristem to flowering is irreversible and leads to the senescence of the inflorescence at seed set. Therefore, restricting the duration of flowering ensures that meristems formed on axillary branches are maintained in the vegetative state and are available later for vegetative growth and further rounds of reproduction. The negative effects of meristem limitation on growth and reproduction have been described previously [16]. In *A. alpina*, reactivation of *PEP1* after vernalization ensures that any axillary meristems that were not induced to flower during vernalization give rise to vegetative branches until the plant is exposed to a further round of vernalization that induces them to flower [15,23]. Thus, restriction of the duration of flowering ensures that vegetative meristems are available throughout the lifetime of the perennial plant. Therefore, among the *A. alpina* accessions studied here, those that do not restrict the duration of flowering are expected to reproduce continuously, and meristem limitation might reduce survival and reproduction in following years. Conversely, flowering continuously without vernalization might be advantageous in environments with high adult mortality, because it would maximize reproduction of relatively young plants and longevity would be less important under these conditions, as described previously for rapid cycling annuals compared with perennials [47,48].

### (b) Geographical variation in the duration of flowering and selection for seasonal flowering

Considering the high genetic variation in Spain, we propose that the obligate requirement for vernalization and short duration of flowering occurs in all Spanish populations because selection favoured a short duration of flowering after vernalization in this environment. Furthermore, the *pep1-1* mutants, which flower perpetually [14], showed poor survival and seed yield in the Spanish environment. The growing season in northern Spain is relatively long and could therefore be divided into periods of reproductive development and vegetative growth followed by growth arrest during summer. By contrast, the short growing seasons of the French Alps and Scandinavia would not allow extensive vegetative development or growth arrest after flowering. Furthermore, snow melts early in the year at the sites of the Spanish populations, which may reduce water availability during the summer (electronic supplementary material, figure S2). By contrast, in the French Alps and Scandinavia, rainfall is supplemented by snowmelt throughout much of the summer in many *A. alpina* habitats (electronic supplementary material, figure S2). Thus, in the Spanish populations, flowering throughout summer might increase susceptibility to higher temperatures or lower water availability. Selection for pleiotropic effects of active *PEP1* alleles on water use efficiency or seed germination [28,49,50] might also contribute to the obligate requirement for vernalization in Spanish populations.

In contrast to Spanish *A. alpina* accessions, some from the French Alps and Scandinavia flowered throughout the growing season. These phenotypes might arise because the loss of the requirement for vernalization has a neutral effect on performance in these environments. Selection for a requirement for vernalization might be relaxed in these environments, because plants are exposed to low temperatures even in the summer and photoperiod may be a more reliable environmental cue for flowering time. Moreover, the shorter growing seasons in Scandinavia and the Alps provide less opportunity to divide the life cycle into seasonal periods of vegetative or reproductive growth. This hypothesis of conditional neutrality is consistent with the higher survival rate of plants that flower in the first year in France compared with Spain and Germany (figure 5).

Alternatively, there might be a selective advantage to perpetual flowering in these populations, at least in some microhabitats or during some years. Perpetually flowering plants might exhibit higher fitness in suitable environments because they can sustain more than one flowering episode each year (electronic supplementary material, figure S7). French Alpine and Scandinavian accessions showed higher survival rates and flowered in higher proportion in the French Alpine and Scandinavian environments, compared with Spain or the German non-natural site. This suggests that both high altitude (Alps) and high-latitude (Scandinavia) environments provide growth conditions that differ from those in the Spanish mountains or outside the natural distribution area (Germany). Nevertheless, the requirement for vernalization varied among maternal families in most French Alpine and Scandinavian populations, which may reflect temporally or spatially varying selection on this trait. In other species, earlier flowering such as that caused by the loss of a requirement for vernalization has been found to be associated with selective advantages [4]. In perennial *Arabidopsis arenosa*, another member of the Brassicaceae, reduced expression of the *FLC* orthologue caused rapid cycling and allowed the spread of a single lineage into lowland Europe along railway tracks and other exposed sites [26]. Furthermore, in *A. thaliana* early flowering caused by loss of *FLC* expression was found to be under positive selection [51], and early flowering has positive effects on fitness in certain environments [52,53]. Therefore, loss of the requirement for vernalization and perpetual flowering in *A. alpina* might also contribute to fitness under particular circumstances in the Alps and Scandinavia.

In conclusion, Spanish populations stand out from other European populations of *A. alpina* because all of them are obligate vernalization-requiring with short duration of flowering, suggesting strong selection, possibly to terminate reproduction before summer. The data provide a basis for genetic and genomic approaches to identify loci under selection in Spain and to assess their association with short duration of flowering and adaptation to a long growing season. Also, they facilitate studies to examine the effects of life-cycle variation within populations in the French Alps and Scandinavia and to determine whether such variation reflects adaptation to different microhabitats.

## Data Availability

The data generated in this study and the code used for data visualization and analyses are deposited in the Dryad Digital Repository (https://doi.org/10.5061/dryad.7wm37pvvm [40]) and in the electronic supplementary material [54].
